# Geography and Access to Kidney Transplant—Are We Measuring What Matters?

**DOI:** 10.1001/jamanetworkopen.2025.49629

**Published:** 2025-12-01

**Authors:** Goni Katz-Greenberg, Lisa M. McElroy

**Affiliations:** Duke University School of Medicine, Durham, North Carolina.

Living-donor kidney transplant (LDKT) and preemptive KT have emerged as superior treatments for end-stage kidney disease (ESKD), with outcomes that surpass both deceased-donor KT (DDKT) after dialysis and dialysis alone. Racial and ethnic disparities in access to KT have been documented for decades, but mounting evidence suggests that inequities are especially pronounced for preemptive transplantation and LDKT.^1,2^ Understanding and dismantling the structural causes of these gaps is essential to achieve the equity goals embedded in several national efforts, such as the Organ Procurement and Transplantation Network (OPTN) modernization initiative and the Centers for Medicare & Medicaid’s Increasing Organ Transplant Access model.

Disparities in transplant access are multifactorial and exert their effects across multiple levels of influence. Patient-level determinants, such as knowledge of transplantation, health status, insurance, financial resources, and personal beliefs and preferences, intersect with community-level factors, such as access to health care, education, and affordable housing. Minority race and ethnicity often overlap with these broader markers of social deprivation and pose a significant challenge for researchers seeking to develop interventions to improve equity in this area.

Elsewhere in *JAMA Network Open*, Li and colleagues^3^ address this gap by examining community-level disadvantage and access to kidney transplant in the United States between 2015 and 2021. They quantified the adjusted hazard ratios (aHRs) of waitlisting, KT, LDKT, and preemptive KT across tertiles of community deprivation as quantified by the neighborhood disadvantage index. They additionally used interaction terms to quantify the differential associations by race and ethnicity.

Their findings are striking. More than one-third of adults with ESKD lived in highly disadvantaged neighborhoods, and these patients were less likely to be waitlisted (aHR, 0.71; 95% CI, 0.69-0.72), to receive any KT (aHR, 0.89; 95% CI, 0.87-0.92), receive LDKT (aHR, 0.65; 95% CI, 0.62-0.69), or receive preemptive KT (aHR, 0.62; 95% CI, 0.58-0.67). Among racial and ethnic subgroups, Black and Hispanic candidates in high-disadvantage neighborhoods had the lowest likelihood of transplantation of any kind. For Black candidates, the likelihood of receiving an LDKT or a preemptive KT was nearly 80% lower than among White candidates in low-disadvantage neighborhoods (LDKT: aHR, 0.23; 95% CI, 0.21-0.25; preemptive KT: aHR, 0.22; 95% CI, 0.20-0.25). Because the study cohort predates the 2022 national transition to race-neutral estimated glomerular filtration rate (eGFR) calculation, these disparities likely encompass delays in disease recognition and referral linked to prior race-based formulas, further amplifying the observed outcomes associated with neighborhood disadvantage.

This study deepens our understanding of the influence of neighborhood conditions on access to kidney transplant by including a specific focus on preemptive KT and LDKT. The differential outcomes associated with race in the context of neighborhood deprivation remind us that while race and ethnicity may not be causal factors, they continue to exert a measurable influence on access to care. However, due to the limitations of the registry data, important questions remain unanswered. The authors found no difference in associations of neighborhood disadvantage with waitlisting for all KT or preemptive KT based on urbanicity, but this differed for LDKT. Residence in high-disadvantage neighborhoods was associated with reduced access for all outcomes, particularly in the Western regions, and in suburban or rural areas for LDKT. This may be explained by the transportation burden faced by residents in rural areas, and several prior studies have demonstrated the influence of donor financial burden on access to LDKT. However, the findings should be interpreted with caution due to the zip code–level assessment of neighborhood disadvantage, which may not fully characterize critical urban areas of need, including areas of rapid gentrification with associated displacement.^4^ More work is needed within densely populated urban areas that characterize neighborhoods within neighborhoods and incorporate key features of urban living, such as public transportation. Ongoing national policy shifts reinforce the importance of structural solutions. The OPTN’s 2022 requirement to use race-neutral eGFR equations—and the 2023 provision allowing backdating of waiting time for affected candidates—illustrate how revising embedded bias in clinical metrics can yield measurable equity gains. Similar intentional embedding of equity metrics within broader system reforms, such as the OPTN modernization initiative, will be essential to ensure that geographic and social disadvantages are addressed as rigorously as biologic ones.

Overall, addressing disparities in access to transplant is a complex, multifaceted issue that demands our continued attention. As chronic disease rates continue to increase, the public health ramifications of this problem become more apparent and alarming. Center-level factors, such as outreach programs, culturally concordant programs, and participation in paired exchange or voucher programs and use of apolipoprotein L1 (APOL1) genetic screening (which may limit identification of suitable Black living donors and listing of prospective Black recipients) are not accounted for, so it is unclear how transplant centers can overcome challenges faced by patients experiencing social deprivation. Broader systemic issues, such as state-level Medicaid expansion, may also serve as a proxy for the political and social policies that affect other structural factors that support health equity. Use of state all-payer databases may provide useful details related to highly rural states.

Li and colleagues^3^ remind us that geography continues to define opportunity in transplantation. Their study also underscores the ongoing need to incorporate discrete data on social context into transplant centers’ reporting requirements. These data, when incorporated into risk stratification models, may help defray the burden of transplant centers serving highly minoritized or deprived communities and provide much-needed financial support for additional social workers and health-related social needs. To ensure that the possibility of kidney transplantation extends to every neighborhood in the United States, we must translate these insights into action—linking data to accountability and accountability to meaningful structural change.
